# The resilience mediation effect on the relationship of suicide risk and psychological pain in nursing students: Structural equation modelling

**DOI:** 10.1111/jpm.13097

**Published:** 2024-08-16

**Authors:** Zeliha Özkan, Dudu Karakaya

**Affiliations:** ^1^ Ministry of Health Antalya Atatürk Public Hospital, Psychiatric Clinic Antalya Turkey; ^2^ Department of Psychiatric Nursing Akdeniz University Faculty of Nursing Antalya Turkey

**Keywords:** nursing students, psychiatric nursing, psychological pain, resilience, structural equation modelling, suicide

## Abstract

**What is known on the subject?:**

- Suicide is an important health problem that has been common all over the world in recent years.
- Nursing students are a high‐risk group for suicide.
- Factors affecting suicide risk should be investigated and included in psychiatric nursing interventions.
- Psychological pain is a predisposing condition for suicide risk.
- Studies focus on the relationship between suicide risk and psychological pain. Studies examining the moderator factors affecting this relationship are limited.

**What the paper adds to existing knowledge?:**

- By means of the structural equation model, the effect of resilience mediation on the relationship of suicide risk and psychological pain has been shown.
- In the process of going from psychological pain to suicide, resilience takes on a buffer function with its mediating effect, and this process can be prevented by the development of resilience.

**What are the implications for practice?:**

- Resilience can be added as a protective factor for suicide to the practices of psychiatric nursing.
- Initiatives to strengthen resilience can be added to psychiatric nursing practices. In this way, psychological pain and the risk of suicide can be reduced by strengthening resilience.
- Psychological pain can be added as a risk factor for suicide to the practices of psychiatric nursing.
- Within the scope of psychiatric nursing practices, nursing interventions can be structured to assess and reduce psychological pain.
- Interventions on strategies to manage and cope with psychological pain and initiatives to strengthen resilience can be added to suicide prevention programs created for nursing students.

**Abstract:**

**Introduction:**

Nursing students are a high‐risk group for suicide. Psychiatric nurses should investigate risk factors for suicide.

**Aim:**

The aim of this study was to examine the resilience mediation effect in the relationship of suicide risk and psychological pain in nursing students.

**Materials and Methods:**

This cross‐sectional study was conducted between March and May 2021 with 619 students in the Akdeniz University Faculty of Nursing. A Descriptive Information Form, the Suicide Probability Scale, the Psychache Scale and the Brief Resilience Scale were used to collect data. The structural equation model approach was used to examine the resilience mediating effect on the relation between suicide risk and psychological pain, and Path Analysis was performed. The reporting of the study was conducted according to STROBE.

**Results:**

A semi‐mediatory effect of resilience was found between psychological pain and the risk of suicide, and the effect size was determined to be broad (0.57).

**Discussion:**

Resilience can be considered as a protective factor for suicide prevention in psychiatric nursing practices.

**Conclusion:**

Resilience has an effect on the relationship between psychological pain and suicide risk.

**Implications for Practices:**

The results of this study may guide psychiatric nursing practices in reducing and preventing suicide risk.

## 1 INTRODUCTION

Suicide is a global problem in the world. In 2019, more than 700,000 people lost their lives through suicide (World Health Organization, 2019), and the World Health Organization has announced a worldwide target of reducing suicide rates by a third by 2030. Researching the factors affecting the act of suicide and including this in intervention plans have gained importance in the context of measures taken against the number of suicide attempts (World Health Organization, 2021).

University students are at risk for suicide. This is a great cause for concern which should not be overlooked (Lambert et al., 2020). Studies have shown that within this group, nursing students are at greater risk of suicide than other university students (Aradilla‐Herrero et al., 2014; Mintz‐Binder, 2007). In a study considering the suicide risk of nursing students, it was concluded that 14% of the students had thoughts of suicide, 14% could be evaluated as being at a high level of suicide risk and 6.5% had a history of attempted suicide (Aradilla‐Herrero et al., 2014). Also, in a study by Moraes et al. (2021), it was found that 53.3% of nursing students carried a risk of suicide and 22.67% had a history of suicide. It was found in another study that one in five of nursing and midwifery students had thoughts of suicide at some time in their lives, and that approximately one in 10 had a history of suicide attempts (Arslantaş et al., 2019). Teaching staff should evaluate the level of risk of suicide of nursing students, and direct them to intervention programmes for treatment. In the event of a suicide attempt, it is important both for teaching staff and for students that there should be a ready crisis intervention plan (Mintz‐Binder, 2007). Psychiatric nurses aim to reduce the risk factors for suicide and suicide attempts by providing holistic care to individuals for the improvement of mental health. (Bulechek et al., 2017; Delaney et al., 2018; Herdman & Kamitsuru, 2017).

In his psychosocial suicide theory, which made a large contribution to the field of the explanation and prevention of suicide, Shneidman proposed that the act of suicide could be caused by the presence of psychological pain, and that psychological pain was one precondition for the risk of suicide (Shneidman, 1999). Other studies have reached conclusions confirming Shneidman's suicide theory (Campos et al., 2019; Ducasse et al., 2018; Lambert et al., 2020; Montemarano et al., 2018). In a study in which it was found that a high level of mental well‐being reduced suicide risk and psychological pain, it was emphasized that it was necessary to evaluate the levels of psychological pain and suicide risk by psychiatric nurses before removing risky behaviours (Tanrıverdi et al., 2024). Another factor playing a determining role in suicide risk is resilience. Psychological resilience can be defined as the capacity to successfully overcome and adapt to negative events such as stress and trauma (Min et al., 2015; Rossetti et al., 2017). A low level of resilience is related to increased risk of suicide (Liu et al., 2014; Rossetti et al., 2017; Roy et al., 2007), and high psychological resilience shows a protective effect against suicide (Min et al., 2015; Ohana et al., 2014).

**FIGURE 1:**
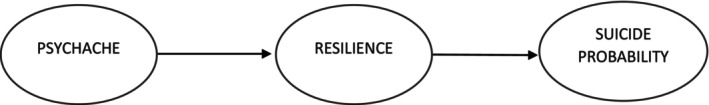
Basic structural model.

Resilience is an important factor which can be used in interventions planned to prevent and treat psychiatric disorders and to preserve mental health (Connor & Zhang, 2006; Davydov et al., 2010). In a systematic review by Shahram et al. (2021), it was emphasized that a primary method of preventing suicide in young people was to increase resilience. Creating and increasing resilience (Ivbijaro et al., 2019) and showing the different aspects of the relation between psychological pain and resilience (Ohana et al., 2014) may be of benefit in preventing suicide. In support of this, it was emphasized in a bibliometric analysis by Cheng et al. (2021) that it was necessary to assess the relation of psychological pain to suicide from a positive psychological aspect and to examine the effect of protective factors. It was thought that resilience, a theory assessed in connection with positive psychology, could be a protective factor between psychological pain and suicide risk, and a structural equation model was set up to determine the mediating effect of psychological resilience in this relationship. The structural equation model tested in this study is given in Figure 1. In this regard, the aim of this study was to determine the mediating effect of resilience between psychological pain and the risk of suicide. With this purpose, an answer was sought in the study to the following questions:

- In nursing students, what relation is there between suicide risk, psychological pain and resilience?
- In nursing students, what is the mediating role of resilience in the relation between psychological pain and suicide risk?

Psychiatric nurses, conducting studies to preserve and improve mental health with findings obtained as a result of testing the structural equation model can plan interventions on strengthening resilience to be able to cope with psychological pain. In this way, individuals who can effectively manage psychological pain with the help of strong resilience will overcome the problem and in this way the risk of suicide will be reduced. Including resilience in interventions in preventing suicides in psychiatric nurses and taking psychological pain into the evaluation as a priority can contribute to individualized nursing care. It is also thought that this study will provide teaching staff with information which can be used in crisis intervention programmes developed to prevent suicide in nursing students.

## 2 METHODS

### 2.1 Study design

This research was performed as a cross‐sectional, descriptive and relation‐seeking study (Erdoğan et al., 2014). The data were collected between March and May 2021. Research reporting was carried out according to Strengthening the Reporting of Observational Studies in Epidemiology (STROBE) (Data S1) (von Elm et al., 2008).

### 2.2 Sample and participants

The population of the study consisted of nursing students registered at the Akdeniz University Faculty of Nursing in the academic year 2020–2021, and it was aimed to contact all nursing students who were willing to take part in the research. The research was conducted with 619 out of 992 nursing students, a participation rate of 62.40%.

### 2.3 Data collection

Education was being conducted online because of the pandemic, and so Google Forms was used to collect data online. An informed consent form, a descriptive information form and the other scales used were created online using Google Forms. First, class representatives were contacted, and the researchers were included in student WhatsApp groups. Then, the students were given information by WhatsApp on the aim and coverage of the research, and the link to the scales created on Google Forms was shared. Each participant took part in the study by entering through a Google account, which prevented more than one entry through the same account. Completion of the data collection forms took approximately 20 min.

### 2.4 Measurements

#### 2.4.1 Descriptive information form

This form was created by the researchers, and consisted of 11 questions on the students' age, gender, marital status, living places, employment status, scholarship status and satisfaction with the university and faculty.

#### 2.4.2 Suicide Probability Scale

The Suicide Probability Scale was developed by Cull and Gill (1989) to assess suicide risk in adolescents and adults, and is a self‐reporting scale of 36 Likert‐type items. The scale consists of four factors: hopelessness, suicidal ideation, poor self‐evaluation and hostility. The highest score which can be obtained on the scale is 144, and the lowest is 36. A high score indicates a high probability of suicide. The validity and reliability study in Turkey was conducted by Batıgün and Şahin (2018), the Cronbach α value was found to be .87. In this study, the Cronbach α value was .93.

#### 2.4.3 Psychache Scale

This 13‐item self‐reporting scale was developed by Holden et al. (2001). The scale is structured according to Shneidman's definition of psychological pain (psychache), and was created to research the relation between psychological pain and tendency to suicide. The results of the scale show the frequency of an individual's psychological pain rather than its intensity. A high score indicates a high level of psychological pain (Holden et al., 2001; Shneidman, 1999). The validity and reliability study in Turkey was conducted by Demirkol et al. (2018), and the Cronbach α value was found to be .98. In this study, the Cronbach α value was .94.

#### 2.4.4 Brief Resilience Scale

This is a 6‐item, 5‐way Likert type scale, developed by Smith et al. (2008) to determine the level of resilience. A high score indicates that the individual has a high level of resilience. The validity and reliability study in Turkey was conducted by Doğan (2015) with university students, and the Cronbach α value was found to be .83. In this study, the Cronbach α value was .85.

### 2.5 Data analysis

For the statistical analysis of data in this study, the programs SPSS (Statistical Package for Social Science) for Windows 23.0 and LISREL 8.71 (Linear Structural Relations) were used. Before beginning the analysis, an examination was made for missing values, outliers and normality. No missing values or outliers were found in the data, and all data were included in the analysis.

In order to determine the mediating effect of resilience between psychological pain and the probability of suicide, first, a structural model was set up, and then Path Analysis was conducted. Before Path Analysis, three measurement models, the psychological pain measurement model, the resilience measurement model and the suicide risk measurement model were defined and tested for the three latent variables included in the model (Table 1, Data S2) (Çokluk et al., 2018; Jöreskog & Sörbom, 1993; Kline, 2005; Tabachnick & Fidell, 2001). The adaptive values of the three measurement models, obtained according to the basic model, were evaluated taking the criteria of fit indices and cut‐off points for acceptance in the structural equation model (Çokluk et al., 2018). It was determined that generally, the fit indices met the acceptance value conditions to a great extent.

**TABLE 1:** Fit indices of measurement models.

Fit indices	SBχ^2^	SD	*p*	SBχ^2^/SD	RMSEA	NNFI	CFI	SRMR	GFI	AGFI
Psychache measurement model	295.27	63	.000	4.69	0.077 (0.068; 0.086)	0.98	0.99	0.037	0.92	0.88
Resilience measurement model	11.49	7	.118	1.64	0.032 (0.000; 0.064)	1.00	1.00	0.019	0.99	0.98
Suicide probability measurement model	10.14	2	.006	5.07	0.081 (0.037; 0.13)	0.98	0.99	0.018	0.99	0.96

In the basic structural model, three implicit variables were defined. These were psychological pain, resilience and probability of suicide. In this model, psychological pain was an exogenous variable, and resilience was an endogenous and at the same time mediating variable. Finally, the probability of suicide was an endogenous variable.

Both in Path Analysis and in testing the measurement models, maximum likelihood analysis was used. Because data deviated from the normal somewhat but at a tolerable level, analyses were performed on the tetrachoric covariance matrix in order to obtain the Satorra–Bentler chi‐square value. To compare hypotheses in dual correlations, Pearson correlation was calculated (Büyüköztürk, 2019; Tabachnick & Fidell, 2001). The internal consistency reliability of the scales used in this study was tested by calculating the Cronbach αvalues. The significance level in the study was taken as .05. In determining effect size, the effect size reference intervals recommended by Cohen (1988) (d) were taken as a base.

### 2.6 Ethics statement

Ethics committee approval for the research was obtained from the Clinical Research Ethics Committee of Akdeniz University (Date: 11 November 2020, Decision No: KAEK‐844), and institutional permission was obtained from the Akdeniz University Faculty of Nursing. Participants were informed about the aim of the research, they were told that their participation was voluntary and their permission was obtained by the use of an informed consent form. Permission was obtained by email from the authors of the scales to be used in the research.

## 3 RESULTS

### 3.1 Sample characteristics

A total of 619 participants were contacted in the research. Their socio‐demographic characteristics are given in Table 2.

**TABLE 2:** Sociodemographic characteristics of nursing students (*n* = 619).

Sociodemographic characteristics	*n*	%
Gender		
Female	456	73.7
Male	163	26.3
Age (years)		
18	35	5.7
19	106	17.1
20	158	25.5
21	156	25.2
22	94	15.2
≥23	70	11.3
Year at school		
First	157	25.4
Second	150	24.2
Third	150	24.2
Fourth	162	26.2
Marital status		
Married	4	0.6
Unmarried	615	99.4
Living places		
With family	463	74.8
Dormitory/apartment	102	16.5
Student house	54	8.7
Employment status		
Employed	44	7.1
Not employed	575	92.9
Scholarship status		
Yes	446	72.1
No	173	27.9
Satisfaction with the faculty		
Satisfied	479	77.4
No satisfied	140	22.6
Satisfaction with the university		
Satisfied	549	88.7
No satisfied	70	11.3
Is your mother alive?		
Yes	612	98.9
No	7	1.1
Is your father alive?		
Yes	595	96.1
No	24	3.9

### 3.2 Correlations between variables

A high‐level positive correlation was found between the nursing students' suicide risk scores and their psychological pain scores (*r* = .738, *p* < .01), and there was found to be common variance between these two variables at a level of 55% (R^2^ = .55). A negative correlation at a medium level was found between suicide risk scores and resilience scores (*r* = −.522, *p* < .01), and the common variance between these two variables was found to be at a level of 27% (R^2^ = .27). It was determined that there was a medium‐level negative correlation between psychological pain scores and resilience scores (*r* = −.530, *p* < .01), and that there was common variance at a level of 28% between these two variables (R^2^ = .28) (Table 3).

**TABLE 3:** The relationship between suicide probability, psychache and resilience in nursing students (*n* = 619).

Variables	Mean	Min/max	Suicide probability	Psychache	Resilience
Suicide probability	32.58 ± 16.07	4/86	1	0.738**	−0.522**
Psychache	28.54 ± 10.48	13/65	.738**	1	−0.530**
Resilience	19.2 ± 4.44	6/30	−.522**	−0.530**	1

** *p* < .01.

### 3.3 Assessment of the structural equation model

**FIGURE 2:**
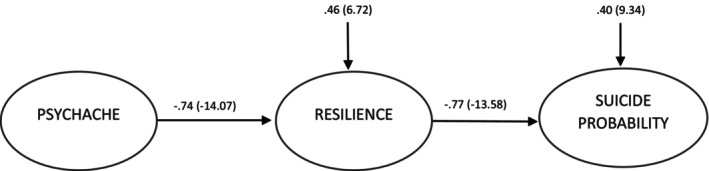
Basic model path coefficients and *t* values.

The path coefficients and *t* values obtained as a result of the path analysis conducted in the research are given in Figure 2. In the path model, both path coefficients are significant. Fit indices relating to whether or not the path model was confirmed are presented in Table 4. The fit indices of the model generally met the acceptance value conditions of the fit indices to a large extent.

**TABLE 4:** Path coefficients and fit indices for the path model.

	*β* coefficient	*t* value
Psychache → Resilience	−.74	−14.07
Resilience → Suicide probability	−.77	−13.58
Fit indices		
SB*χ* ^2^		845.41
*p*		.000
SD		224
*χ* ^2^/SD		3.77
RMSEA		0.067 (0.062; 0.072)
SRMR		0.082
GFI		0.88
NNFI		0.98
CFI		0.98

### 3.4 Mediation correlation in the path model

It was found that resilience had a semi‐mediating effect between psychological pain and suicide risk, and the effect size was determined to be large (.57) (*t*:14.96) (Table 5).

**TABLE 5:** Mediation correlation in the path model.

	*β* ^a^	*β* ^b^	Effect size	*t*	Conclusion
Psychological pain → Resilience → Suicide probability	−.61/−.20	.68	0.57	14.96	The effect size large and a semi‐mediation effect

^a^ Beta coefficient for direct effects.

^b^ Beta coefficient for indirect effects.

## 4 DISCUSSION

In this study, which was conducted with the aim of investigating the mediating effect of resilience on the relation between psychological pain and suicide risk in nursing students, it was concluded that resilience had a semi‐mediating effect and that the effect size was large. Also, the analyses showed that there were significant correlations between the variables of psychological pain, suicide risk and resilience, confirming the literature. It is thought that a new viewpoint has been gained for the literature on preventing suicide in accordance with the mediating effect of resilience. The information obtained as a result of this research is important in preventing and reducing suicide.

It was found in the study that resilience showed a semi‐mediating effect between psychological pain and suicide risk. In a bibliometric analysis by Cheng et al. (2021) examining 230 articles between 1994 and 2020 on the relation between suicide and psychological pain, it was recommended that in future studies, the relation of psychological pain to suicide risk should be assessed from the point of view of positive psychology, and the effect of protective factors should be examined. It is thought that with this study, the importance has been shown of resilience, one of the important concepts of positive psychology, in the connection between the risk of suicide and psychological pain. In the conclusion of the research, it was shown that resilience had a protective effect in the relation of the probability of suicide and psychological pain, and could be assessed as a mediating variable. In other words, it can be said that resilience can be seen to have a buffer function with its semi‐mediating effect between psychological pain and suicide. In this way, the psychological process leading to suicide can be controlled by the protective effect of resilience.

The model shows a significant correlation between suicide risk and psychological pain. It is seen in the literature that an increase in the level of psychological pain is paralleled by an increase in the risk of suicide. In addition, it is emphasized that psychological pain must be assessed and reduced in order to largely weaken other risk factors of suicide (Berardelli et al., 2020). Studies have shown that psychological pain has a greater effect on the risk of suicide than other factors associated with suicide risk such as depression and hopelessness (Calati et al., 2020; Frumkin et al., 2021; Lambert et al., 2020; Levi et al., 2008; Montemarano et al., 2018; Reisch et al., 2010; Troister et al., 2015). It was found in one review examining suicide tendency in university students that psychological pain was one of the important variables for educators to reduce the risk of suicide in students. It has been emphasized that even after hopelessness and depression have been alleviated, psychological pain is still an important factor which can raise the risk of suicide (Javed & Munawar, 2021). It was seen in the model that this correlation was confirmed by current literature on nursing students. In this regard, it can be said that psychological pain can be assessed as a precursor when establishing the risk of suicide.

The process of successfully coping with and accommodating to negative events which individuals encounter can be explained by resilience (Arslan, 2015; Gizir, 2007; Karaırmak, 2006). Also, positive life experiences and positive thought are used to strengthen psychological resilience (Seligman & Csikszentmihalyi, 2000). Positive things such as social support, academic success, supportive parents, positive relationships, a positive social life, being happy and getting satisfaction from life are protective factors which can be used to improve resilience (Arslan, 2015; Dearden, 2004). Suicide risk can be reduced by strengthening psychological resilience with the effect of these factors (Rossetti et al., 2017). It has been shown that high resilience in individuals has a protective effect against suicidal thoughts (Min et al., 2015). Studies have found that resilience levels were low in people with a history of suicide attempts, and this has been associated with an increase in suicide risk (Knowles et al., 2021; Rossetti et al., 2017). In a study conducted with nursing students, it was concluded that low self‐esteem and low resilience increased the risk of suicide (Montes‐Hidalgo & Tomás‐Sábado, 2016). In a systematic literature scan, it was concluded that a primary way of preventing suicide in young people was resilience (Shahram et al., 2021). The results of this study support findings obtained in the current literature, and show that low resilience is correlated with the risk of suicide.

An important factor driving people to suicide is the desire to escape from psychological pain, and strong coping mechanisms have an effect on this (Campos et al., 2019). It can be said in this regard that the relation between resilience and psychological pain can be important for the risk of suicide. In the literature, a higher level of psychological stability has been associated with lower psychological pain (Ohana et al., 2014; Zhang et al., 2022). It has been stated that psychological stability is a protective factor for psychological pain (Zhang et al., 2022). Similar to the literature, it was also found in this study that there was a negative correlation between resilience and psychological pain. In this regard, it can be said that psychological pain can be reduced by strengthening resilience. Resilience can be thought of as a strong motivational component in escaping unbearable psychological pain.

Faculty members and teaching staff are responsible for providing nursing students who are at risk of suicide with access to the resources which they need (Stubin, 2020). Studies have shown that nursing students should be assessed for the risk of suicide and intervention programmes should be created for at‐risk students (Aradilla‐Herrero et al., 2014; Moraes et al., 2021), and that there is a need for a previously arranged crisis intervention plan to be implemented in the case of a possible suicide attempt (Mintz‐Binder, 2007). Also, in a study by Heath et al. (2021), it was seen as a result of a programme implemented to improve resilience in nursing students that increased resilience raised the state of mental well‐being, and could be helpful in preventing suicides. In this regard, interventions on strategies to manage and cope with psychological pain and initiatives to strengthen psychological resilience can be added to crisis intervention and suicide prevention programmes created for students.

Psychological pain can be added as a risk factor and resilience as a protective factor to the practices of psychiatric nursing. Evaluation of psychological pain and nursing interventions to reduce it can be constructed. Initiatives to strengthen resilience can be added to psychiatric nursing practices. In this way, psychological pain and the risk of suicide can be reduced by strengthening resilience. It is thought that according to the result obtained from the structural equation model, nursing interventions can be constructed in line with the semi‐mediating effects of resilience.

## 5 LIMITATIONS

This study was limited to students of Akdeniz University Faculty of Nursing, and the results cannot be generalized as they rely on self‐reporting. The findings of this study can only be generalized to its participants. Also, since it is a cross‐sectional study, a cause–effect relationship cannot be established.

## 6 CONCLUSION

By testing the structural equation model created in this study, it was concluded that resilience had a semi‐mediating effect between psychological pain and the probability of suicide, and that the effect size was large. The analyses conducted showed that the variables of psychological pain, risk of suicide and resilience were significantly correlated with each other. It is thought that these results can show the way to psychiatric nursing practices to reduce suicide risk. Because the study group was a group of young adults, the results can be integrated into intervention programmes for groups at risk of suicide, and can be helpful in preventing suicide. Also, studies can be planned with individuals with mental problems taking into account the correlation of suicide risk, psychological pain and resilience and testing the structural model. Other factors which are thought to be related to suicide risk and psychological pain can be added to the model as mediating factors and tested. It is recommended that self‐efficacy and self‐esteem, which are concepts which may be related to suicide risk and psychological pain, be tested by being added to the structural model as mediating variables. It is predicted that the concept of self‐efficacy, defined as a person's belief in their ability to manage and cope with events, may be effective in that person's ability to cope with psychological pain and suicide risk. In addition, it is thought that self‐esteem, consisting of the three aspects of self‐love, self‐acceptance and competence, will be able to affect the response to psychological pain and have a psychological effect on the process which leads to suicide.

## 7 RELEVANCE STATEMENT

With this study, it is thought that important evidence that will guide interventions for reducing the risk of suicide and preventing suicide has been determined and a roadmap for suicide prevention has been presented. The results of this study reveal the importance of increasing psychological resilience in reducing psychological pain and preventing suicide. Since the sample is a young adult group, the results of this study can be integrated into intervention programmes for groups at risk for suicide.

## FUNDING INFORMATION

This research did not receive any specific grant from funding agencies in the public, commercial or not‐for‐profit sectors.

## CONFLICT OF INTEREST STATEMENT

No conflict of interest has been declared by the author(s).

## Supporting information


Data S1.

Data S2.

## Data Availability

Data available on request from the authors.
