# Supplementary material for: The resilience mediation effect on the relationship of suicide risk and psychological pain in nursing students: Structural equation modelling
Source: J Psychiatr Ment Health Nurs. 2024 Aug 16;32(1):172–80. doi: 10.1111/jpm.13097 (PMC11704997; doi:10.1111/jpm.13097)
Supplement: Supplementary file 2 — Data S2. [file JPM-32-172-s002.docx]

**Supplementary Material-2**

**PSYCHACHE MEASUREMENT MODEL**

**Psychache measurement model t values**

**Psychache measurement model standardized coefficients and error variances**

**RESILIENCE MEASUREMENT MODEL**

**Resilience measurement model t values**

**Resilience measurement model standardized coefficients and error variances**

**SUICIDE PROBABILITY MEASUREMENT MODEL**

**Suicide Probability measurement model t values**

**Suicide Probability measurement model standardized coefficients and error variances**
